# Employment among Patients with Multiple Sclerosis-A Population Study

**DOI:** 10.1371/journal.pone.0103317

**Published:** 2014-07-23

**Authors:** Hanne Marie Bøe Lunde, Wenche Telstad, Nina Grytten, Lars Kyte, Jan Aarseth, Kjell-Morten Myhr, Lars Bø

**Affiliations:** 1 Department of Neurology, Norwegian Multiple Sclerosis Competence Centre, Haukeland University Hospital (HUS), Bergen, Norway; 2 Department of Neurology, Sogn and Fjordane Central Hospital, Førde, Norway; 3 Faculty of Health Studies, Sogn and Fjordane University College, Førde, Norway; 4 Department of Clinical Medicine, KG Jebsen Center for MS research, University of Bergen, Bergen, Norway; 5 Department of Neurology, Norwegian Multiple Sclerosis Registry and Biobank, Haukeland University Hospital, Bergen, Norway; San Raffaele Scientific Institute, Italy

## Abstract

**Objective:**

To investigate demographic and clinical factors associated with employment in MS.

**Methods:**

The study included 213 (89.9%) of all MS patients in Sogn and Fjordane County, Western Norway at December 31^st^ 2010. The patients underwent clinical evaluation, structured interviews and completed self-reported questionnaires. Demographic and clinical factors were compared between patients being employed versus patients being unemployed and according to disease course of MS. Logistic regression analysis was used to identify factors independently associated with current employment.

**Results:**

After a mean disease duration of almost 19 years, 45% of the population was currently full-time or part- time employed. Patients with relapsing –remitting MS (RRMS) had higher employment rate than patients with secondary (SPMS) and primary progressive (PPMS). Higher educated MS patients with lower age at onset, shorter disease duration, less severe disability and less fatigue were most likely to be employed.

**Conclusions:**

Nearly half of all MS patients were still employed after almost two decades of having MS. Lower age at onset, shorter disease duration, higher education, less fatigue and less disability were independently associated with current employment. These key clinical and demographic factors are important to understand the reasons to work ability in MS. The findings highlight the need for environmental adjustments at the workplace to accommodate individual ’s needs in order to improve working ability among MS patients.

## Introduction

Multiple Sclerosis (MS) is a chronic debilitating central nervous system (CNS) illness that is associated with a high unemployment rate in early adulthood [1]. Inflammation, demyelination and axonal damage are pathological hallmarks giving rise to the characteristic multifocal CNS lesions seen in MS [2]. The symptoms that come along with having MS reflect the multifocal nature of the pathology, by showing a wide individual variation and severity. In dealing with the unpredictable nature of disease progression, the individual affected is left with a high degree of uncertainty about future occupational demands and work ability. The school-to-work transition may pose particular challenges for MS patients who are physically disabled or have a cognitive dysfunction. MS is one of the leading causes of non-traumatic disability affecting young adults in Europe and the USA, and the degree of physical disability has shown to be a strong predictor of work ability [3]–[5]. Non-motor symptoms like pain, fatigue and memory impairment as well as demographic factors such as age and educational background have also shown significant impact on employment status in MS [6], [7]. Thus, employment may be regarded as a marker of overall functioning of the individual patient, and have also important impact on quality of life (QoL) [8]. Several studies have investigated and described demographic and clinical features associated with employment status in different cohorts of MS patients [9]–[11]. However, we are not aware of any studies that have investigated employment status in a county based MS population and its subsequent clinical subtypes: relapsing- remitting MS (RRMS), secondary progressive MS (SPMS) and primary progressive MS (PPMS). To further explore MS patient’s ability to work, we therefore investigated demographic and clinical factors influencing employment status in a population based MS cohort from Sogn and Fjordane County, Western Norway.

## Materials and Methods

### Ethics Statement

Written informed consent was obtained by all the participants and the study was approved by the local regional committee for research ethics, Western Norway.

### Patients

The study was conducted during 2008–2010 at the Central Hospital of Sogn and Fjordane County in the city of Førde, Western Norway. All MS patients in the county are diagnosed, registered and receive regular follow-up visits at the Department of Neurology at this Hospital. All prevalent MS patients in the county at December 31^st^ 2010 were invited to participate in an extensive study of the clinical and demographic impact of MS. A total of 213 of 237 (89.9%) MS patients in Sogn and Fjordane County consented for participation and were available for inclusion in the study. The patients fulfilled the diagnostic criteria of McDonald, and were classified according to disease course into RRMS, SPMS and PPMS. Marital status, education, disease course, onset symptoms, chronic pain and disability were registered or scored at time of interview and examination. All other data were obtained from patient self-assessment questionnaires. Demographic and clinical data were compared between subtypes of MS and between patients being employed versus patients being unemployed. Written informed consent was obtained by all the participants and the study was approved by the local regional committee for research ethics, Western Norway.

### Employment status

Current employment status was recorded, dichotomized and defined as employed (full time and part time) or not employed. Full time work for Norwegian employees is defined as 37.5 hour per week. Reasons for not being employed were divided into six different categories: sick leave; unemployment; disability pension; retirement pension; education and others. Type of occupation was registered and classified into two categories based on manual physical strength; light physical work (administration, teacher, secretary) and heavy physical work (nurse, craftsman, farmer etc.) [12], [13]. Age and gender specific employment rates of the general population in Sogn og Fjordane County in 2010 were obtained from Statistics Norway, and compared to the employment rates of the MS patients.

### Demographic characteristics of patient population

We recorded demographic data on age, gender, education and marital status. Education was recorded and categorized into lower educational levels ≤12 years (primary, secondary and high school) and higher educational levels >12 years (college, university).

### Clinical characteristics

Year and symptoms of onset, disease course categorized as RRMS, SPMS and PPMS, fatigue, disability, presence of chronic pain, depression and use of disease modifying treatment (DMT) were recorded.

Onset symptoms were initially categorized into seven groups according to Kurtzke’s Functional Systems; visual, sensory, brainstem, cerebellar, pyramidal, bowel/bladder and others, but were further merged into three groups (visual-sensory, brainstem-cerebellar, pyramidal) due to small numbers.

Fatigue was assessed by the Fatigue Severity Scale (FSS), which is one of the most widely used fatigue severity specific questionnaires in MS [14]. The Norwegian version of the FSS is validated and has previously shown satisfactory psychometric properties [15].

The patients were clinically examined, and neurological disability score was registered by use of the Expanded Disability Status Scale (EDSS) [16].

Chronic pain was recorded as constant or intermittent pain experienced every day over the past 3 months. For analytical purposes, pain was dichotomized into present or not, regardless of localization or type of pain.

Depression was assessed according to the original Beck Depression Inventory (BDI) [17]. This score range from 0–63 and scores 0–9 are considered normal, scores 10–19 as mild depression, scores 20–29 as moderate depression and scores from 30–63 as severe depression. According to Kendall, AT Beck and co-workers in “Issues and recommendations regarding use of the Beck depression inventory” the term depression are recommended used for patients with scores from 20–30 (moderate depression) and patients with scores from 10–19 may be referred to as “dysphoric”. Based on the BDI score, the patients were therefore categorized as not being depressed (score 0–19) and depressed (score 20–63) [18].

Anxiety was assessed according to Beck Anxiety Inventory (BAI) [19]. This score range from 0–63 and scores 0–15 are considered minimal or mild anxiety and scores from 16–25 as moderate anxiety and scores from 26–63 as severe anxiety. Based on the BAI scores, the patients were therefore categorized as not having anxiety (scores 0–15) and having anxiety (scores 16–63) [19].

Type of disease modifying treatment (DMT) (interferon beta, glatiramer acetate, natalizumab, fingolimod, mitoxantrone) was analyzed as present use or not, but not further classified, due to small group sizes.

### Statistical analysis

For continuous variables following a normal distribution One -Way analysis of variance (ANOVA) was used to compare different types of MS, age at onset and disease duration. Non parametric test of Kruskal-Wallis was applied for ordinal and continuous variables of non-normality (time from onset to diagnosis, fatigue and disability). Fisher exact test was used to compare categorical variables (gender, married/cohabitant, education, onset symptoms, depression, anxiety, chronic pain and disease modulating therapy). Shapiro –Wilk test in combination with inspection of the normal Q-Q plots were used to assess the assumption of normality.

We used a binary regression model to estimate odds ratios (OŔs) in order to identify possible independent factors associated with the outcome variable (being employed). Building a regression model included several steps as described by Hosmer and Lemeshow [18]. The selection process of covariates related to employment began with a univariate analysis. Any variable with P<0.25 was selected as a candidate for the multivariate model based on the Wald test [20]. More traditional levels of p<0.05 can fail in identifying variables known to be important [20]. All candidates were included in a model and one by one non-significant variables were removed until the final model containing all significant variables. In this process large changes in OR for the remaining variables were thoroughly inspected. The Hosmer-Lemeshow goodness of fit test was used to test good fit for our regression model. Further, cross-tabulation and analysis of missing data in the multivariate analysis with respect to age at onset, married status and gender were performed, and there were no significant differences recorded between missing data to the cases included in the analysis. Statistical significance was defined as p≤0.05, two tailed tests. The data were analysed by using SPSS software for Windows (IBM SPSS Statistics21).

## Results

### Demographic and clinical characteristics in MS; RRMS, SPMS and PPMS

All 213 MS patients were available for socio-demographic analysis; among them 147 women and 66 men, giving a female: male ratio of 2.2∶1 (Table 1). The initial courses were RRMS in 186 patients, of whom 112 still had RRMS, whereas 74 patients had developed SPMS. A total of 27 patients had PPMS. Mean age of the cohort was 49.8 (±12.0) years, and significant differences between RRMS (43.2±9.9), SPMS (55.8±10.1) and PPMS (60.9±7.9) were revealed (p<0.001) (data not shown). The mean disease duration for the entire cohort was 18.9 (±11.5) years and significant variations according to subtype of MS were noted; RRMS (13.9 years), SPMS (27.3 years), PPMS (16.9 years) (p<0.001). Median time from onset to MS-diagnosis for the whole cohort was 4.0 years and we found distinct differences between subtypes of MS; RRMS (3.0 years), SPMS (7.0 years) and PPMS (2.0 years) (p = 0.001). There were no differences regarding marital status (p = 0.29) or levels of education (p = 0.37) between MS subtypes.

**Table 1:** Socio-demographic and clinical characteristics in multiple sclerosis (MS); Relapsing-remitting MS, Secondary progressive MS and primary progressive MS.

Variables	Total population (n = 213)	RRMS (n = 112)	SPMS (n = 74)	PPMS (n = 27)	*P* value	Missing observations#
**Age at onset, yrs, mean (±SD)** *	32.7 (±9.9)	31.2 (±9.0)	30.4 (±8.0)	45.6 (±8.3)	<0.001	0
**Time from onset to diagnosis,** **yrs, median (IQR)** ***	4.0 (±8.0)	3.0 (±7.0)	7.0 (±8.0)	2.0 (±5.0)	0.001	0
**Disease duration, yrs, mean (± SD)** *	18.9 (±11.5)	13.9 (±9.4)	27.3 (±10.8)	16.9 (±7.8)	<0.001	0
**Gender** **						
** Female, n (%)**	147 (69.0)	84 (75.0)	52 (70.3)	11 (40.7)	0.002	
** Male, n (%)**	66 (31.0)	28 (25.0)	22 (29.7)	16 (59.3)	0.002	0
**Married/cohabitant, n (%)** **	155 (72.8)	85 (75.9)	49 (66.2)	21 (77.8)	0.29	0
**Education** **						
**More than 12 years (>), n (%)**	62 (29.1)	36 (32.1)	21 (28.4)	5 (18.5)	0.37	0
**Onset symptoms** **						
**Visual-sensory (yes), n (%)**	98 (50.5)	56 (50.0)	39 (52.7)	3 (11.1)	<0.001	0
**Brainstem-cerebellar (yes), n (%)**	50 (26.0)	31 (27.7)	15 (20.3)	4 (14.8)	0.27	0
**Pyramidal (yes), n (%)**	36 (18.5)	19 (17.0)	9 (12.2)	8 (24.6)	0.12	0
**Fatigue (FSS), median (IQR)** ***	5.3 (±2.0)	5.0 (±2.0)	5.6 (±2.0)	6.1 (±2.0)	0.061	39
**Disability (EDSS), median (IQR)** ***	4.0 (±3.0)	3.0 (±2.0)	6.0 (±3.5)	5.5 (±2.8)	<0.001	17
**Depression (present; BDI-D), n (%)** **	11 (6.5)	8 (8.9)	3 (5.1)	0 (0.0)	0.30	44
**Anxiety (present; BDI-A), n (%)** **	32 (18.7)	16 (17.4)	14 (23.3)	2 (10.5)	0.41	42
**Chronic pain (present) n (%)** **	95 (55.9)	57 (60.0)	28 (50.0)	10 (52.6)	0.47	16
**Disease modulating** **therapy (yes), n (%)** **	56 (27.1)	44 (40.4)	10 (14.1)	2 (7.4)	<0.001	6

^*^P-values (crude) were calculated from analysis of variance (ANOVA), α = 0.05.

^**^P-values (crude) were calculated from chi-square analysis (Fischer exact test), α = 0.05.

^***^P-values (crude) were calculated from Kruskal Wallis; α = 0.05.

Missing observations#; n (number of missing values) ranging from 0–44.

FSS: Fatigue Severity Scale; EDSS: Expanded Disability Status Scale; BDI: Beck Depression Inventory, RRMS: relapsing-remitting MS, SPMS: secondary progressive MS, PPMS: primary progressive MS.

Both frequency of visual/sensory onset symptoms (p = 0.001) and mean level of neurological disability (p<0.001) differed significantly among subtypes of MS (Table 1). As expected, more frequent use of disease modifying treatment (DMT) was registered in patients with RRMS than in patients with progressive types of MS (p<0.001). About 55.9% (95/197) of patients reported having chronic pain, but no variation according to subtypes of MS was seen (p = 0.47). More patients reported presence of anxiety (18.7%) than depressive (6.5%) symptoms, without differences between MS subtypes (p = 0.41, p = 0.30). Only ten patients received antidepressants, of which 9 used selective serotonin reuptake inhibitor and one a tricyclic agent.

### Employment rate among MS patients and the general population

Employment among MS patients was compared to the employment rates in the general population of Sogn and Fjordane County by age and gender groups at December 31^st^ in 2010 (Table 2). Only 33% of male and 32% of female MS patients aged 55–66 were employed compared to 75% of men and 69% of women in the general population.

**Table 2:** Employment rates (full time and part time) in 2010 among the general population and MS-patients in Sogn og Fjordane County.

Age groups	General Population Employment rates (%)		MS population Employment rates (%)	
	men	women	men	women
20–24 years	71.9	68.0	100	100
25–39 years	87.5	82.9	58.3	68.8
40–54 years	90.6	87.8	44.0	51.6
55–66 years	75.0	68.8	33.3	32.4

### Univariate analyses of factors associated with employment in MS

A total of 213 patients were available for evaluation of employment status (full-time and part-time) (Table 3). The employment rate among the patients was forty five percent (45.1%), of whom 19.2% patients were full-time employed. Part-time employees constituted 25.8% of the employed population (RRMS; 33.9%, SPMS; 20.3% and PPMS 7.4%) (p<0.001) (data not shown). About 66% of RRMS patients were full-time employed in comparison with 24.3% of SPMS and 14.8% of PPMS (p<0.001) (Table 3). A significant variation in employment rate between the three EDSS groups (0–3, 3.5–6, >6) was seen, and patients with an EDSS score between 0–3 had the highest employment rate (70.8%) (p<0.001). No significant differences between employed patients versus unemployed patients were seen regarding gender (p = 0.41), marital status (p = 0.97), visual-sensory (p = 0.11), brainstem-cerebellar (p = 0.26) and pyramidal onset symptoms (p = 0.78). Type of occupation (p = 0.26), presence of chronic pain (p = 0.42), depression (p = 0.23) and anxiety (p = 0.18) were not statistically significant in relation to employment (Table 3a). Mean age at onset (p = 0.03), disease duration (p<0.001) and mean score of fatigue (p<0.001) in employed MS patients were significantly lower than in unemployed patients (Table 3). Among patients with higher levels of education; 56.5% of patients were employed compared to 40.4% of patients with lower education (OR 1.91, p = 0.03). Employed patients received disease modulating therapy (DMT) more frequently than non-employed patients (p = 0.02) (Table 3). Of the patients not being employed; 7 patients were on sick leave, two were students, eleven patients received retirement pension and 97 patients received disability pension (data not shown).

**Table 3:** Univariate analysis of factors associated with employment in MS.

Variables	All patients (n = 213)	Employed (n = 96)	Unemployed (n = 117)	OR (95% CI)	P value
**Age at onset, yrs, mean(±SD)** *	32.7(±9.9)	31.1 (±8.9)	34.0 (±10.4)	0.97 (0.94–0.99)	0.03
**Disease duration, yrs, mean (±SD)** *	18.9 (±11.5)	15.1 (±10.0)	22.1 (±11.8)	0.94 (0.91–0.96)	<0.001
**Gender, n (%)** **					
** Male**	66 (31)	27 (40.9)	39 (59.1)	1	0.41
** Female**	147 (69)	69 (46.9)	78 (53.1)	1.28 (0.71–2.30)	
**Marital status, n (%)** **					
** Single**	58 (27)	26 (44.8)	32 (55.2)	1	0.97
** Married/cohabitant**	155 (73)	70 (45.2)	85 (54.8)	0.99 (0.54–1.81)	
**Education, n (%)** **					
** ≤12 years**	151 (71)	61 (40.4)	90 (59.6)	1	0.03
** >12 years**	62 (29)	35 (56.5)	27 (43.5)	1.91 (1.05–3.47)	
**Disease course, n (%)** **					
** RRMS**	112 (52.6)	74 (66.1)	38 (33.9)	1	<0.001
** SPMS**	74 (34.7)	18 (24.3)	56 (75.7)	0.17 (0.09–0.32)	
** PPMS**	27 (12.7)	4 (14.8)	23 (85.2)	0.09 (0.03–0.28)	
**Occupation, n (%)** **					
** Light physical**	106 (50.5)	52 (49.1)	54 (50.9)	1	0.26
** Heavy physical**	104 (49.5)	43 (41.3)	61 (58.7)	0.73 (0.42–1.26)	
**EDSS, categorized, n, (%)** **					
** 0–3**	72 (17)	51 (70.8)	21 (29.2)	1	<.001
** 3.5–6**	91 (43)	36 (39.6)	55 (60.4)	0.27 (0.14–0.52)	
** >6**	33 (40)	2 (6.1)	31 (93.9)	0.027 (0.006–0.12)	
**Fatigue, total (±SD)** *	5.0 (±1.6)	4.5 (±1.5)	5.4 (±1.6)	0.67 (0.54–0.82)	<0.001
**Depression (BDI), n (%)** **					
** No**	158 (93)	73 (46.2)	85 (53.8)	1	0.23
** Yes**	11 (7)	3 (27.3)	8 (72.7 )	0.44 (0.11–1.70)	
**Anxiety (BDI), n (%)** **					
** No**	139 (81)	66 (47.5)	73 (52.5)	1	0.18
** Yes**	32 (19)	11 (34.4)	21 (65.6)	0.58 (0.26–1.29)	
**Chronic pain, n (%)** **					
** No**	75 (44)	37 (49.3)	38 (50.7)	1	0.42
** Yes**	95 (56)	41 (43.2)	54 (56.8)	0.78 (0.44–1.43)	
**Onset symptoms** **					
** Visual-sensory**					
** No**	115 (54)	46 (40.0)	69 (60.0)	1	0.11
** Yes**	98 (46)	50 (51.0)	48 (49.0)	1.56 (0.90–2.69)	
** Brainstem-cerebellar**					
** No**	163 (76.5)	70 (42.9)	93 (57.1)	1	0.26
** Yes**	50 (23.5)	26 (52.0)	24 (48.0)	1.44 (0.76–2.71)	
** Pyramidal**					
** No**	177 (83)	79 (44.6)	98 (55.4)	1	0.78
** Yes**	36 (17)	17 (47.2)	19 (52.8)	1.11 (0.54–2.27)	
**MS course at onset** **					
** RRMS**	186 (87)	91 (48.9)	95 (51.1)	1	0.005
** PPMS**	27 (13)	5 (18.5)	22 (81.5)	0.24 (0.08–0.66)	
**Disease modulating therapy**					
** No**	151 (73)	61 (40.4)	90 (59.6)	1	0.02
** Yes**	56 (27)	33 (58.9)	23 (41.1)	2.12 (1.13–3.95)	

*P-values (crude) were calculated from analysis of variance, a = 0.05.

**P-values (crude) were calculated from chi-square analysis (Fischer exact test), a = 0.05.

### Multivariate analyses of factors associated with employment in MS

Multivariate analysis revealed that lower age at onset (OR = 0.93, p = 0.004), shorter disease duration (OR = 0.93, p = 0.020), higher education (OR = 2.28, p = 0.048) less fatigue (OR = 0.73, p = 0.011) and lower level of neurological disability (p = 0.001) were independently associated with employment (Table 4). The goodness of fit test had a p-value equal 0.97 demonstrating a good fit for our model. A total of 172 (80.7%) patients were included in this final model.

**Table 4:** Multivariate analysis of factors associated with employment in MS*.

Variables	OR (95 %CI)	P value**
**Age at onset, yrs, OR (95 % CI)**	0.93(0.89–0.98)	0.004
**Disease duration, yrs, OR (95 % CI)**	0.93 (0.89–0.98)	0.02
**Education, OR ( 95 % CI)**		
** ≤12 years**	1	0.048
** >12 years**	2.28 (1.01–5.17)	
**EDSS, categorized, OR ( 95 % CI)**		
** 0–3**	1	0.001
** 3.5–6**	0.34 (0.15–0.77)	
** >6**	0.05 (0.01–0.26)	
**Fatigue OR ( 95 % CI)**	0.73 (0.57–0.93)	0.011

*n = 172.

**p value≤0.05 calculated from binary regression analysis.

## Discussion

This study set out with the aim of evaluating employment status in a county based MS population and to investigate characteristic demographic and clinical features in MS-subtypes. One notable finding was the clear differences in employment rate in the various subtypes of MS. In the present study we found that 66.1% of patients with RRMS were employed full-time or part –time. In comparison, only 24.3% of SPMS patients and 14.8% of PPMS patients were employed. These findings are in line with two former studies, showing that patients with RRMS with lower disability levels have a higher employment rate than patients with progressive types of MS with more pronounced disabilities [21], [22].

Epidemiological differences between RRMS and PPMS were further identified in our cohort. In the present study, more males (59%) than females were affected by PPMS, corresponding to previous reports on gender distribution in PPMS [23]. Patients with PPMS were older at onset, had predominately motor symptoms at onset, had higher disability levels and were more frequently unemployed compared to patients with RRMS and SPMS. These results are largely consistent with a previous descriptive study on PPMS in Northern Ireland and UK [24].

Binary logistic regression analysis showed the importance of disease duration on employment. The average disease duration of employed MS patients in the present study was 7 years shorter than among unemployed patients. Patients with RRMS had a mean disease duration of 13.9 years, approximately half that of SPMS at 27.3 years, and less than in PPMS; 16.9 years. The impact of disease duration on employment longevity has been shown by others [25], [26]. Reportedly, between 50–80% of unemployed MS patients have left work within 10 years post diagnosis [26]. Nonetheless, at an average disease duration of nearly 19 years, almost half (45%) of the MS population were still employed either full- time or part- time, a somewhat larger proportion than in other similar studies [12], [27].

In the present study, lower age at onset was independently associated with employment, confirming previous studies [3], [9], [13]. MS patients with a current working engagement were approximately three years younger at initial onset of MS than unemployed patients. Also; an average 10 year age gap separated the employed MS patients from the unemployed patients. This discrepancy in mean age between employed patients contra unemployed patients may reflect legislative protection or flexibility of working practices that facilitate young employees with chronic diseases in Norway. Also, there are welfare benefits awarding middle aged residents with chronic diseases in Norway that may motivate patients to leave workforce early. The national welfare benefits may explain why age plays a different role in studies of employment in MS.

We could not detect any independent relation of disease modulating therapy on employment, confirming a previous study from UK (5) [28]. However a short –term prospective study has indicated some beneficial effects on employment (30). Our study included a cross-sectional population based cohort of patients with a mean disease duration of almost 20 years, implying that some patients have not received any treatment at all, and others may have received treatment in more advanced stages of the disease. Thus a prospective study design would have been preferable to study DMT effects on employment.

The disability levels in MS were lower in RRMS. We found less severe disability to be independently associated with current employment. These results are consistent with other studies [3], [4], [22]. Fifty seven percent of employed MS patients had EDSS scores of three or less (fully ambulatory without aid). The likelihood of being employed varied significantly according to EDSS scores. MS patients with an EDSS score between 0–3 had a three- fold likelihood of being employed compared to MS patients with an EDSS score ranging from 3.5–6. In general, an EDSS score above 5 is associated with marked preclusion of daily activities [16]. Our results are consistent with a multinational MS survey that found stable MS to be the main reason among employed MS patients for remaining at work [29].

In the present study less severe fatigue was independently associated with employment. Univariately, employed MS patients had an average score of 1.0 point lower FSS than unemployed patients. Fatigue has also been found to play an important role related to changes in occupational status in numerous previous studies [3], [4], [7], [10], [21], [30], and was rated as the commonest factor associated with leaving the workforce prematurely [29]. Fatigue is also the factor MS patients themselves believe to cause their own unemployment [1]. This could possibly be explained by the frequent presence of fatigue in MS, but also to its debilitating effects on both physical and mental health.

Highly educated patients had more than a twofold risk of being employed compared to patients with less education. The onset of a chronic illness at an early age may be disruptive to the education process. In MS, however, disease onset is usually between 30–40 years of age, at an age where most patients would have completed their education and started a working career. Higher education is associated with better job opportunities and a greater likelihood of being employed at all with better employment benefits and higher wages [31]. In addition to economic benefits, higher education represents social advantages including sense of control, social standing and greater influence over own working conditions [32]. Higher educated individuals are also more likely to practice health promoting behaviors such as to abstain from smoking and attend regular health check-ups [32]. All reasons that could explain the higher risk of employment among highly educated MS patients.

Type of occupation (heavy versus light physical job) did not influence employment status among the MS patients. This finding is in accordance with two previous studies that also found occupation types to be unrelated to employment [11], [12]. However, none of these studies, including ours, examined other important working conditions like flexible work schedules and other important workplace accommodations. Numerous working conditions in addition to jobs demanding physical strength were examined in Verdier-Taillefer et al. case-control study on factors related to unemployment among MS patients, and this study found that jobs requiring physical strength increased the odds of unemployment [21].

The present study was a cross-sectional cohort study, meaning that we could not predict causality between work ability and the different variables studied. In addition, future studies should also address issues not pursued in our study such as cognitive functional tests to assess possible relations between cognitive disability and employment. In addition, we did not assess environmental work factors that could influence employment, such as support by co- workers and close family, the ability to rest at work and the relation between employer and employee at the work place.

We began this research with the purpose of gaining a better understanding of the reasons related to work ability in MS. From descriptive analyzes, we observed a significantly higher employment rate among patients with RRMS than in patients with progressive types of MS, PPMS in particular. The final multivariate results showed that highly educated MS patients with lower age at onset, shorter disease duration and less fatigue and disability were more likely to be employed. These findings provide important insight and understanding of the underlying demographic and disease specific factors related to employment opportunity in MS. This suggests the need for legislative practice and environmental adjustments at the workplace to improve working ability among less educated, fatigued and disabled MS patients.
